# Effect of statin on long-term outcomes in persistent tobacco users receiving percutaneous coronary intervention: A longitudinal, retrospective cohort study

**DOI:** 10.1097/MD.0000000000040463

**Published:** 2024-11-08

**Authors:** Mao-Jen Lin, Hau-De Lin, Chuan-Zhong Cai, Ming-Jen Chuang, Feng-Ching Yang, Kuo Feng Chiang, Han-Ping Wu

**Affiliations:** a Department of Medicine, Taichung Tzu Chi Hospital, The Buddhist Tzu Chi Medical Foundation, Taichung, Taiwan; b Department of Medicine, College of Medicine, Tzu Chi University, Hualien, Taiwan; c Department of Pediatrics, Chang-Gung Medical Foundation Chiayi, Chang-Gung Memorial Hospital, Chiayi County, Taiwan; d College of Medicine, Chang Gung University, Taoyuan, Taiwan.

**Keywords:** all-cause mortality, percutaneous coronary intervention, smoking, stain

## Abstract

The role of statins in improving cardiovascular outcomes is well established, but little is known about their impacts on long-term outcomes in persistent tobacco users with stable coronary artery disease (CAD) who receive percutaneous coronary intervention (PCI). A population of persistent smokers with CAD treated by PCI was analyzed. From 2012 through 2019, a cohort of 907 persistent tobacco users with stable CAD undergoing PCI were enrolled from the inpatient department of Taichung Tzu Chi Hospital, Taiwan. We surveyed statin users and non-statin users after index PCI, and general characteristics, major risk factors, angiographic findings, and long-term clinical outcome were compared. Kaplan–Meier curve was used to compare the survival difference and Cox proportional hazard model was used to analyze the predictors for all-cause mortality and major adverse cardiovascular events, including cardiovascular (CV) mortality, myocardial infarction, and repeated PCI procedures. The statin group had a higher average total cholesterol (*P* < .01) and low-density lipoprotein cholesterol (LDL-C) level (*P* < .01) and was younger (*P* < .01) than the non-statin group. Ninety-six point one percent patients in the statin group had a LDL-C level below 100 mg/dL after treatment. They also had a more frequent history of acute coronary syndrome and lower prevalence of chronic kidney disease than the non-statin group (both *P* < .01). Freedom from all-cause and CV mortality were lower in the non-statin group than the statin group (both *P* < .01). After adjustment for age and chronic kidney disease, statin treatment no longer reduced the risk of CV mortality (hazard ratio: 0.32, 95% confidence interval = 0.07–1.49), but was still associated with a reduction in all-cause mortality (hazard ratio: 0.27, 95% confidence interval = 0.10–0.75). In persistent tobacco users undergoing PCI, patients treated with statin for LDL-C values above 100 mg/dL had a similar level of cardiovascular protection as those with LDL-C below 100 mg/dL and without statin treatment. Therefore, smoking attenuates pleiotropic effect of statin. Nevertheless, statin therapy was still associated with a reduction of all-cause mortality.

## 1. Introduction

Percutaneous coronary intervention (PCI) is a very important therapeutic strategy in patients with stable coronary artery disease (CAD) in addition to medical treatment. However, the impact of persistent tobacco use in patients with stable CAD receiving PCI remains controversial. Smoking might lead to advanced coronary atherosclerosis and impaired microvascular function, putting patients at higher risk of future adverse outcomes.^[1]^ Previous studies reported a significantly increased risk of cardiovascular (CV) events, including mortality, in current smokers with stable CAD compared to never-smokers.^[2]^ Smoker’s paradox refers to a controversial phenomenon of an unexpected favorable outcome of smokers after acute myocardial infarction (MI). Possible explanations include smokers had better response to thrombolysis due to the more frequent thrombotic nature of coronary artery occlusion, presumably caused by a smoking-related hypercoagulable state; in contrast with the often more critical residual coronary stenoses found in nonsmokers. Besides, baseline difference such as smokers were younger and they had significantly lower rates of traditional CV risk factors might also lead to favorable outcomes.

In patients undergoing PCI, the data is conflicting. Some study concluded smoking has no protective effect on 6-month clinical outcomes compared to never-smokers.^[3]^ However, 1 recent study found the smoking paradox might still exist for long-term prognosis in patients with stable CAD undergoing PCI.^[4]^ Tobacco use is a major risk factor for coronary atherosclerosis and the mechanism is complex. Exposure to tobacco consumption might activates a serial mechanisms predisposing to atherosclerosis, including thrombosis, increase insulin resistance and dyslipidemia, abnormal vascular growth, and angiogenesis, vascular inflammation, as well as loss of endothelial cell functions.^[5–7]^ The chemical components in tobacco might lead to progression of atherosclerosis. Among them, nicotine, polycyclic aromatic hydrocarbons, oxidizing agents have been identified as potential contributing factors to atherogenesis. Nicotine might accelerate vascular disease through inducing catecholamines release, nicotine also increases blood pressure and heart rate. Besides, nicotine-induced catecholamine release increases platelet aggregability. All of these negative hemodynamic effects are associated with progression of atherosclerosis. Enhanced platelets aggregation might lead to the growth of atherosclerotic plaque via the accretion of thrombus, and through the release of growth factors which induce vascular smooth muscle cell proliferation.^[8]^ Cigarette smoking will lead to progression of atherosclerosis based on ultrasound findings of increased intima-media thickness of carotid artery through 3-year follow-up.^[9]^

Statin treatment has been demonstrated to improve long-term CV outcomes in patients with acute coronary syndrome (ACS).^[10,11]^ Furthermore, high-dose statin therapy might provide more clinical benefits than low-dose statin therapy.^[12–15]^ Statins might also reduce major adverse cardiovascular events (MACE) in patients with coronary microvascular dysfunction.^[16,17]^ On the other hand, persistent tobacco use might attenuate the beneficial effect of statin.^[18]^ Among persistent tobacco users with stable CAD who have undergone PCI, differences in long-term outcomes of those with average low-density lipoprotein cholesterol (LDL-C) levels who are not receiving a statin compared to those with elevated LDL-C levels who are receiving a statin remain unclear, and there is a paucity of data on the subject. We hypothesize persistent tobacco use will negate the cardiovascular protection effect provide by statin therapy among patients with stable CAD undergoing PCI. Therefore, the focus of current study was to compare long-term outcomes between patients with statin therapy to those without statin therapy, and to analyze the adverse predictors for long-term clinical outcomes in persistent tobacco users with stable CAD undergoing PCI.

## 2. Materials and methods

### 2.1. Study population

From 2012 through 2019, this longitudinal, retrospective cohort study enrolled about 1400 persistent tobacco users with stable CAD aged 20 to 90 years from the inpatient department of Taichung Tzu Chi Hospital, Taiwan. Survey of medical records and coronary angiography were completed. All patients were divided into 2 groups: on statin treatment and non-statin statement. Not until 2019, the guideline for statin usage conducted by Taiwan National health Insurance Administration, Ministry of Health and Welfare only allows use statin to treat patients with CAD with a LDL-C level above 100 mg/dL and the goal is LDL-C <100 mg/dL. Those with LDL-C level below 100 mg/dL are not allow to be used.^[19]^ According to the guideline provided by Taiwan National Health Insurance Administration, Ministry of Health and Welfare, statin was prescribed in participants with a LDL-C level above 100 mg/dL and the goal is LDL-C <100 mg/dL in current study. Patients with stage PCI, NYHA class IV heart failure, MACE occurred within 1 month after index PCI, and underlying malignancy were all excluded. Patients received regular follow-up through the outpatient department, but for patients lost to follow-up a telephone call was used to contact patients or their families. The Institution Review Board and ethics committee of Taichung Tzu Chi Hospital approved the study protocol (REC111-18) and informed consent was waived from all patients due to its retrospective design. The methods used in current study accorded with the relevant clinical guidelines and regulations. This cohort study also fulfilled the guidance of the Strengthening the Reporting of Observational Studies in Epidemiology (STROBE) statement.^[20]^

### 2.2. Data pooling, measurement, and analysis

Data collected included baseline general characteristics, including age, sex, body mass index, and biochemical data; hemodynamic data and coronary angiography obtained during catheterization; major risk factors; therapeutic approach such as medications prescribed after index PCI; and intervention strategies, including balloon angioplasty, bare-metal stent deployment, or drug-eluting stent (DES) deployment. The definitions of never smoker and current smokers are according to the U.S. Centers for Disease Control.^[21]^ Never smoker is defined as someone who has smoked < 100 cigarettes over their lifetime, whereas current smoker is defined as someone who has smoked 100 cigarettes over his or her lifetime and who also currently smokes cigarettes. After index PCI procedure, intervention for quitting smoking including oral persuasion offered by attending physician and instruction list for abstaining smoking will give to participant himself or families. Only patients who were current smokers both at enrollment and at the last follow-up were included in the study as persistent tobacco users. The types and dosage of statins used in this study were also recorded. Conventional definition of major risk factors was used in current study and has been previously described.^[22–24]^ Diabetes mellitus was defined as either a fasting plasma glucose level of more than 126 mg/dL, a casual plasma glucose level >200 mg/dL or a hemoglobin A1c level of more than 6.5%; hypercholesterolemia was defined as a LDL-C level more than 100 mg/dL or a serum total cholesterol level of more than 200 mg/dL; hypertension was defined as a systolic blood pressure (SBP) ≥ 140 mm Hg or diastolic blood pressure (DBP) ≥ 90 mm Hg according to JNC 7 classification; chronic kidney disease (CKD) was defined as an estimated glomerular filtration rate of <60 mL/min/1.73 m^2^, which is equal to or more than stage III CKD. As for cardiac intervention, left ventriculography via contrast injection during cardiac catheterization or nuclear ventriculography was used to evaluate the systolic function. Coronary angiography was reviewed and interpreted, including lesion anatomy, number of diseased vessels and lesions. Lesion severity and complexity was calculated via Synergy between PCI with Taxus and cardiac surgery score (SYNTAX score).^[25]^ General characteristics, exposed risk factors, catheterization findings, and types of invasive approach were compared. Primary end-points were all-cause mortality and MACE including CV mortality, MI and clinically driven repeated PCI procedures over the study period. The beginning time was the date of index PCI and the duration was from its beginning through December 2020 or the date of any primary endpoint occurred.

### 2.3. Statistical analysis

The analysis was primarily used to compare the differences between the 2 groups. Continuous variables was examined by independent *t* tests whereas categorical variables was examined by chi-square test or Fisher exact test. The log-rank test and Kaplan–Meier curves were used for comparing survival differences. The Cox proportional-hazards model was used to test the effect of independent variables on hazards. A *P* value of <.05 was considered significant. All analyses were performed using SPSS for Windows, Version 23.0 (IBM Corp., Armonk, NY).

## 3. Results

### 3.1. General characteristics

**Figure 1.**
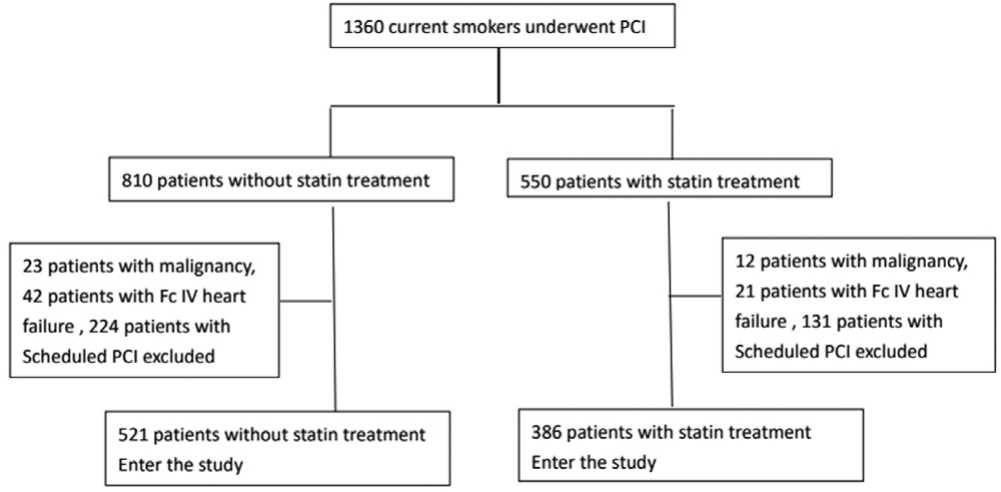
Algorithm for study population enrollment.

Over the 4-year study period, 907 persistent tobacco users with stable CAD who received a successful PCI procedure were recruited, and a flow chart of the algorithm for participant’s enrollment was shown in Figure 1. Among them, 521 patients were in the non-statin treatment group while 386 patients were in the statin treatment group. Statin treatment were continued after index PCI unless serious adverse event happened. In the statin treatment group, 151 patients used atorvastatin (39.1%, median dose: 20 mg), 197 patients used rosuvastatin (51%, median dose: 10 mg), 23 patients used pitavastatin (6.0%, median dose: 2 mg), and 15 patients used simvastatin (3.9%, median dose: 20 mg). At the end of study, the mean cholesterol and LDL-C level in statin treatment group are 151 ± 24.3 mg/dL and 75 ± 28.7 mg/dL, respectively, 96.1 % patients in statin group had a LDL-C level below 100 mg/dL. Follow-up periods for the non-statin treatment group and the statin treatment group were 44.4 ± 37.2 month versus 47.1 ± 33.1 months (*P* = .27). General characteristics of the study groups are listed in Table 1. Statin treatment patients were younger than non-statin treatment patients (58.0 ± 11.9 vs 62.1 ± 12.3 years-old, *P* < .01), they also had a higher body mass index than non-statin treatment patients. (26.1 ± 3.9 vs 25.5 ± 4.0 kg/m^2^, *P* < .03). Statin treatment group had a lower central pulse pressure (CPP) than non-statin treatment patients (57.2 ± 18.6 vs 60.1 ± 21.5 mm Hg, *P* = .03). As for baseline biochemistry, the statin treatment group had a higher average total cholesterol level than the non-statin treatment group (194.0 ± 43.8 vs 168.0 ± 38.6 mg/dL, *P* < .01); however, the statin treatment group had a significantly elevated LDL-C level than the non-statin treatment group (124.4 ± 39.0 vs 98.9 ± 32.7 mg/dL, <0.01).

**Table 1:** Demographic and metabolic characteristics of the study population.

Variable	Study groups		*P* value
	Statin (N = 386)	Non-statin (N = 521)	
Age (years)	58.0 ± 11.9	62.1 ± 12.3	<.01*
Weight (kg)	72.9 ± 11.4	69.8 ± 12.8	<.01*
Height (m)	1.67 ± 0.06	1.65 ± 0.07	<.01*
BMI (kg/m^2^)	26.1 ± 3.9	25.5 ± 4.0	.03*
CSP (mm Hg)	132.4 ± 22.5	133.9 ± 24.8	.33
CDP (mm Hg)	75.2 ± 13.2	73.6 ± 13.7	.09
CPP (mm Hg)	57.2 ± 18.6	60.1 ± 21.5	.03*
Cholesterol (mg/dL)	194.0 ± 43.8	168.0 ± 38.6	<.01*
HDL (mg/dL)	37.6 ± 15.5	38.1 ± 15.5	.62
TG (mg/dL)	159.9 ± 103.5	155.1 ± 117.2	.51
LDL (mg/dL)	124.4 ± 39.0	98.9 ± 32.7	<.01*
Creatinine (mg/dL)	1.6 ± 4.9	1.9 ± 2.3	.23

BMI = body mass index, CDP = central diastolic pressure, CPP = central pulse pressure, pulse pressure represents the difference between systolic and diastolic blood pressure, CSP = central systolic pressure, HDL = high-density lipoprotein cholesterol, LDL = low-density lipoprotein cholesterol, TG = triglyceride.

* Significant.

### 3.2. Demographic and clinical profiles

Demographic and clinical profiles for the study population are shown in Table 2. In terms of DM and hypertension, there was no prevalence difference between statin treatment group and non-statin treatment group (P = NS). The statin treatment group had a higher percentage of previous MI history (*P* < .01), whereas the non-statin treatment group had a higher prevalence of CKD and previous history of stroke (both *P* < .01). As for medication used after index PCI, we found the statin treatment group used aspirin, a P2Y12 inhibitor (*P* < .01), beta-blockers (BB) (*P* < .01), and angiotensin receptor blocker (*P* = .02) more frequently than the non-statin treatment group, while the non-statin treatment group had a higher rate of fibrate usage (*P* < .01) compared to statin treatment group.

**Table 2:** Risk factors and medications prescribed after index PCI in groups.

Variable	Study groups		*P* value
	Statin (N = 386)	Non-statin (N = 521)	
Gender			.64
F	14 (3.6%)	16 (3.1%)	
M	372 (96.4%)	505 (96.9%)	
Hypertension			.66
No	167 (43.3%)	233 (44.7%)	
Yes	219 (56.7%)	288 (55.3%)	
DM			.10
No	270 (70.0%)	337 (65.0%)	
Yes	118 (30.4%)	182 (35.0%)	
Previous ACS			<.01*
No	189 (49.0%)	337 (64.7%)	
Yes	197 (51.0%)	184 (35.3%)	
CKD			<.01*
No	279 (72.3%)	306 (58.7%)	
Yes	107 (27.7%)	215 (41.3%)	
Stroke history			<.01*
No	377 (97.7%)	484 (92.9%)	
Yes	9 (2.3%)	37 (7.1%)	
CABG history			.75
No	385 (99.7%)	519 (99.6%)	
Yes	1 (0.3%)	2 (0.4%)	
Aspirin			<.01*
No	15 (3.9%)	64 (12.3%)	
Yes	371 (96.1%)	457 (87.7%)	
P2Y12 inhibitors			<.01*
No	21 (5.4%)	72 (13.8%)	
Yes	365 (94.6%)	449 (86.2%)	
Diuretics			.19
No	320 (82.9%)	414 (79.5%)	
Yes	66 (17.1%)	107 (20.5%)	
BB			<.01*
No	172 (44.6%)	288 (55.3%)	
Yes	214 (55.4%)	233 (44.7%)	
CCB			<.01*
No	294 (76.2%)	355 (68.1%)	
Yes	92 (23.8%)	166 (31.9%)	
ACEI			.12
No	306 (79.3%)	434 (83.3%)	
Yes	80 (20.7%)	87 (16.7%)	
ARB			.02
No	258 (66.8%)	386 (74.1%)	
Yes	128 (33.2%)	135 (25.9%)	
Fibrate			<.01*
No	379 (98.2%)	470 (90.2%)	
Yes	7 (1.8%)	51 (9.8%)	

ACEI = angiotensin-converting enzyme inhibitor, ARB = angiotensin receptor blocker, BB = beta-blockers, CABG history = history of coronary artery bypass graft, CCB = calcium channel blocker, CKD = chronic kidney disease, DM = diabetes Mellitus, HC = hypercholesterolemia, P2Y12 inhibitor = P2Y12 receptor inhibitor of platelet, Previous ACS = previous acute coronary syndrome.

* Significant.

**Figure 2.**
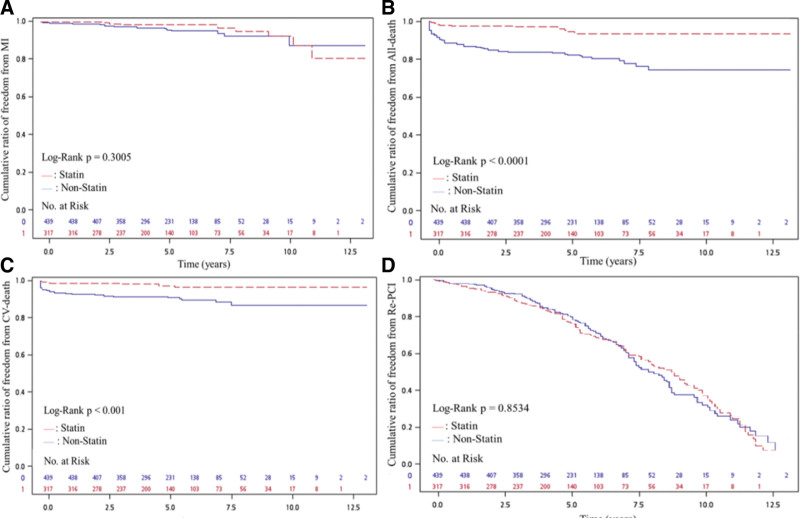
(A) Cumulative ratio of freedom from MI between 2 groups (*P* = .301). (B) Cumulative ratio of freedom from all-cause death between 2 groups (*P* < .001). (C) Cumulative ratio of freedom from CV death between 2 groups (*P* < .001). (D) Cumulative ratio of freedom from Re-PCI between 2 groups (*P* = .8534). CV death = cardiovascular death, MI = myocardial infarction, Re-PCI = repeated percutaneous coronary intervention.

Results of the catheterization findings and major outcomes are list in Table 3. Based on coronary angiography, the number of diseased vessels, treated vessels and lesions, or SYNTAX scores were not significant different between the 2 groups. However, balloon angioplasty was performed more frequently in the non-statin treatment group, while the statin treatment group had a higher rate of DES (mainly second generation) deployment (*P* < .01). All-cause mortality and CV mortality were higher in the non-statin treatment group compared with the statin treatment group (both *P* < .01), but there is no statistical significance between the 2 groups as for MI and repeated PCI procedures. Figure 2 reveals the cumulated rate of freedom from MI, all-cause mortality, CV mortality and repeated PCI procedures between the 2 groups. Base on the Kaplan–Meier survival curve, freedom from CV mortality and all-cause mortality and were lower in the non-statin treatment group compared with the statin treatment group (both *P* < .001), but there were no differences for MI (*P* = .30) or repeated PCI procedures (*P* = .85).

**Table 3:** Angiographic findings and outcome according to statin use.

Variable	Study groups		*P* value
	Statin (N = 386)	Non-statin (N = 521)	
Follow-up time (months)	44.4 ± 37.2	47.1 ± 33.1	.27
Number of diseased vessel			.05
Single vessel disease	158 (40.9%)	245 (47.0%)	
Dual vessel disease	140 (36.3%)	150 (28.8%)	
Triple vessel disease	88 (22.8%)	126 (24.2%)	
Mean of treated vessels	1.3 ± 0.5	1.3 ± 0.5	.28
Mean of treated lesions	1.6 ± 0.8	1.5 ± 0.8	.26
SYNTAX score	12.4 ± 8.0	11.4 ± 8.4	.09
LVEF (%)	56 ± 14	56 ± 13	.49
Type of intervention			<.01*
Balloon angioplasty	74 (19.2%)	153 (29.4%)	
BMS deployment	166 (43.0%)	238 (45.7%)	
DES deployment	201 (52.1%)	200 (38.4%)	
MI			.07
Yes	9 (2.3%)	17 (3.3%)	
No	377 (97.7%)	504 (96.7%)	
CV death			<.01*
Yes	9 (2.3%)	41 (7.9%)	
No	377 (97.7%)	480 (92.1%)	
All-cause death			<.01*
Yes	14 (3.6%)	75 (14.4%)	
No	372 (96.4%)	446 (85.6%)	
Re-PCI			.37
Yes	137 (35.5%)	170 (32.6%)	
No	249 (64.5%)	351 (67.4%)	

BMS = bare-metal stent, CV death = cardiovascular death, DES = drug-eluting stent, DM = diabetes mellitus, HC = hypercholesterolemia, LVEF = left ventricular ejection fraction, MI = myocardial infarction, Re-PCI = repeated percutaneous coronary intervention, SYNTAX score = Synergy Between PCI With Taxus and Cardiac Surgery score.

* Significant.

### 3.3. Clinical outcomes

Outcomes analysis from the Cox proportion hazard model, presented with hazard ratios (HR) and 95% confidence intervals (CI) before and after adjustment for MI, all-cause mortality, CV mortality, and repeated PCI procedures, is listed in Table 4. Syntax score predicted MI before (HR: 1.07, 95% CI: 1.07–1.12) and after (HR: 1.14, 95% CI: 1.04–1.18) adjustment. Before and after adjustment, age (HR: 1.05, 95% CI: 1.02–1.08 vs HR: 1.05, 95% CI: 1.02–1.09) and SYTNAX score (HR: 1.04, 95% CI: 1.01–1.07 vs HR: 1.04, 95% CI: 1.01–1.08) predicted all-cause mortality, while previous stroke history predicted CV mortality after adjustment (HR: 5.27, 95% CI:1.25–22.14). Statin usage reduce all-cause mortality before (HR: 0.22, 95% CI: 0.09–0.53) and after (HR: 0.27, 95% CI: 0.10–0.75) adjustment. Statin usage seemed to reduce CV mortality before adjustment (HR: 0.20, 95% CI: 0.06–0.62); however, it was no longer significant after adjustment of age and CKD (HR: 0.32, 95% CI: 0.07–1.49). Finally, we found that usage of P2Y12 inhibitors predicted repeated PCI procedures before (HR: 1.86, 95% CI: 1.10–3.14) and after (HR: 1.86, 95% CI: 1.09–3.15) adjustment, whereas prescription of angiotensin-converting enzyme inhibitor could reduce repeated PCI procedures before (HR: 0.60, 95% CI: 0.42–0.85) and after (HR: 0.54, 95% CI: 0.37–0.77) adjustment.

**Table 4:** Significant outcome predictors for MI, all-cause death, CV death, and repeated PCI procedures in Cox proportion hazard model.

Variables	MI		All-cause mortality		CV mortality		Repeated PCI	
	Crude HR (95% CI)	Adjusted HR (95% CI)	Crude HR (95% CI)	Adjusted HR (95% CI)	Crude HR (95% CI)	Adjusted HR (95% CI)	Crude HR (95% CI)	Adjusted HR (95% CI)
Age	0.99 (0.94–1.04)	0.95 (0.89–1.01)	1.05 (1.02–1.08)**	1.05 (1.02–1.09)**	1.03 (0.99–1.07)	0.98 (0.93–1.03)	1.00 (0.98–1.01)	0.99 (0.98–1.01)
DM	1.09 (0.37–3.19)	1.63 (0.54–4.94)	1.25 (0.67–2.34)	1.21 (0.61–2.40)	1.34 (0.60–2.99)	0.63 (0.24–1.71)	1.26 (0.91–1.75)	1.21 (0.87–1.67)
ACS	1.86 (0.68–5.09)	1.06 (0.35–3.17)	2.43 (1.29–4.57)**	1.16 (0.59–2.28)	3.52 (1.44–8.59)**	1.91 (0.75–4.86)	1.22 (0.89–1.67)	1.26 (0.92–1.73)
CKD	1.54 (0.44–5.33)	1.11 (0.23–5.34)	1.00 (0.47–2.14)	1.59 (0.70–3.60)	0.80 (0.29–2.19)	1.27 (0.39–4.18)	1.19 (0.81–1.76)	1.08 (0.73–1.60)
Stroke	–	–	1.15 (0.43–3.06)	0.90 (0.30–2.64)	1.78 (0.56–5.71)	5.27 (1.25–22.14)*	1.43 (0.68–3.04)	1.64 (0.77–3.51)
Syntax score	1.07 (1.02–1.12)*	1.11 (1.04–1.18)**	1.04 (1.01–1.07)*	1.04 (1.01–1.08)*	1.06 (1.02–1.10)**	1.03 (0.99–1.09)	1.00 (0.98–1.02)	1.00 (0.98–1.02)
DES	1.05 (0.36–3.08)	1.08 (0.30–3.85)	0.59 (0.30–1.16)	0.63 (0.29–1.34)	0.66 (0.29–1.52)	0.97 (0.34–2.75)	1.08 (0.78–1.49)	1.09 (0.79–1.50)
Aspirin	–	–	2.06 (0.73–5.83)	1.07 (0.36–3.22)	4.47 (0.59–33.80)	0.72 (0.07–6.98)	1.82 (0.95–3.48)	1.63 (0.85–3.13)
P2Y12 inhibit	1.86 (0.23–15.01)	1.46 (0.16–13.43)	1.75 (0.66–4.61)	1.67 (0.56–4.98)	2.61 (0.58–11.73)	1.29 (0.19–8.69)	1.86 (1.10–3.14)*	1.86 (1.09–3.15)*
BB	1.75 (0.66–4.62)	2.46 (0.76–7.76)	0.43 (0.23–0.81)**	0.49 (0.24–1.00)	0.38 (0.17–0.86)*	1.61 (0.45–5.75)	1.13 (0.84–1.52)	1.12 (0.83–1.50)
CCB	1.69 (0.55–5.15)	0.90 (0.25–3.16)	0.55 (0.25–1.22)	0.55 (0.24–1.29)	0.54 (0.18–1.63)	0.30 (0.06–1.37)	1.01 (0.71–1.43)	0.98 (0.69–1.38)
ACEI	1.42 (0.50–4.01)	1.05 (0.31–3.57)	0.59 (0.29–1.22)	0.66 (0.30–1.41)	0.64 (0.26–1.58)	0.88 (0.30–2.61)	0.60 (0.42–0.85)**	0.54 (0.37–0.77)**
Statin	0.54 (0.19–1.56)	0.53 (0.16–1.70)	0.22 (0.09–0.53)**	0.27 (0.10–0.75)*	0.20 (0.06–0.62)**	0.32 (0.07–1.49)	0.86 (0.63–1.17)	0.84 (0.61–1.61)

ACEI = angiotensin-converting enzyme inhibitor, ACS = previous acute coronary syndrome, BB = beta-blockers, CCB = calcium channel blocker, CKD = chronic kidney disease, CPP = central pulse pressure, CV mortality = cardiovascular mortality, DES = drug-eluting stent, DM = diabetes mellitus, MI = myocardial infarction, P2Y12 inhibitor = P2Y12 receptor inhibitor of platelet, SYNTAX score = Synergy Between PCI With Taxus and Cardiac Surgery score.

* *P* < .05.

** *P* < .01.

## 4. Discussion

The CV protection provided by statins seemed to attenuate in persistent tobacco users with stable CAD, but statin usage was still associated with reduction of all-cause mortality. Besides, SYNTAX scores predicted for MI attack in current smokers. Age and SYNTAX score were both predictors of all-cause mortality whether current smokers used statin or not, while statin usage could reduce the hazard of all-cause mortality. Previous stroke history was a predictor for CV mortality in current smokers. Finally, P2Y12 inhibitors were a predictor for repeated PCI procedures, whereas angiotensin-converting enzyme inhibitor reduced the hazard of repeated PCI procedures.

Reducing serum LDL-C level have a positive role of preventing progression of atherosclerosis. There were many randomized clinical trials to support reducing LDL-C to reduce atherosclerotic cardiovascular disease. Statins are the most widely prescribed and evidence-based lipid-lowering drug in the world for lowering LDL-C and reducing CV morbidity and mortality, both in primary and secondary prevention (WOSCOPS, CARDS, CARE, HPS, ASCOT-LLA, etc). Statin can inhibit hepatic cholesterol synthesis, leading to increased production of microsomal HMG-CoA reductase and increased cell surface LDL receptor expression. This facilitates increased clearance of LDL-C from the bloodstream and a subsequent reduction in circulating LDL-C. In addition to reducing LDL-C and cardiovascular morbidity and mortality, statins may have additional pleiotropic effects, which include improvements in endothelial function, stabilization of atherosclerotic plaques, antithrombotic, immunomodulatory, and anti-inflammatory effects.

In current smokers, there were no gender differences, but the average age of the non-statin group was older than the statin group. The CPP during cardiac catheterization was also higher in the non-statin group than the statin group, while elevated CPP had a negative impact on long-term mortality.^[26]^ Although the statin group is younger, statins therapy still have a pleiotropic effect in improving endothelial dysfunction and reducing the atherosclerotic burden, thus reduce arterial stiffness and prevent atherosclerosis progression. The average serum LDL-C level was higher than 100 mg/dL in the statin group, whereas the average serum LDL-C level in the non-statin group was lower than 100 mg/dL, which met with guideline-recommend statin therapy.^[27,28]^ Although statin use when LDL-C levels are between 50 and 125 mg/dL has been proved to improve CV outcomes in CAD patients after ACS,^[29]^ it remains obscure whether statin use could improve clinical outcomes in current smokers with stable CAD undergoing PCI with a serum LDL-C level below 100 mg/dL, further research is warranted to address this issue. Besides, P2Y12 inhibitors (mainly clopidogrel and ticagrelor) are widely used in both group, persistent smoking is well known to enhance CYP1A2 activity, theoretically it could increase the antiplatelet efficacy of these thienopyridine drugs.^[30–32]^ However, routine CYP gene polymorphism detection in current smokers receiving P2Y12 inhibitors is not available in Taiwan, hence the interaction between persistent tobacco use and P2Y12 inhibitors is hardly to be determined. In the other hand, BB were underused in both group, since nicotine-mediated central nervous system activation may diminish the effect of BB on blood pressure and heart rate. Therefore, smokers taking BB might require higher dosages than nonsmokers.^[33]^

While there were no differences in the distribution or number of diseased vessels in the statin and non-stain group, non-statin treatment group had an increased prevalence of triple vessel disease and a trend toward significance (*P* = .05). The number of treated vessels, lesions and SYNTAX Scores were also similar between the 2 groups; however, balloon angioplasty was used more commonly in non-statin group while more DES were used in the statin group. One possible explanation for balloon angioplasty being used more frequently in the non-statin group is their higher prevalent rate of CKD, since excess contrast consumption should be avoid in CKD patients undergoing PCI and simple balloon angioplasty in theory requires less contrast media than subsequent bare-metal stent or DES deployment. Although more DES were deployed in the statin group than the non-statin group, a previously reported post hoc analysis of Chinese patients found that DES deployment did not seem to reduce all-cause death or MACE^[34]^ in current smokers.

In addition to improving coronary microvascular dysfunction and reducing MACE in patients receiving coronary angiography, long-term statin therapy has also been reported to improve epicardial coronary perfusion after PCI.^[35]^ However, persistent smoking might negate the cardiovascular protection provided by statin therapy. In recent study, optical coherence tomography was used to evaluate the impact of smoking on plaque morphological changes in non-culprit lesions in patients 1 year after ACS. It found persistent smokers had a thinner fibrous cap and the incidence of thin-cap fibroatheroma is higher compared with nonsmokers, regardless of statin therapy.^[36]^ Persistent smoking thus might cause plaque instability in patients receiving statin therapy and thus increase the adverse cardiovascular events. This is consistent with findings of current study, which found that statin seems not reduce the hazard of CV mortality. Although the non-statin group was older, thus lead-time bias might exist and they had a higher prevalence of CKD than the statin group, we found statins still reduced all-cause mortality after adjustment for age and CKD. Statin treatment has been proved to reduce all-cause mortality in patients with nonobstructive CAD,^[37]^ yet the role of statins in obstructive CAD is less clear. Nevertheless, current guidelines emphasize the importance of smoking cessation in all CAD patients, regardless of whether they have received PCI or coronary artery bypass graft.

Many limitations still exist in current study. First, the medical intensity, such as strict blood glucose, blood pressure and lipid control, were not scrutinized in this study, which may confound the results. Second, pack-years of smoking were not fully evaluated in this study, which might relate to severity of coronary atherosclerosis and thus influence long-term outcome. Third, the number of MI events in both groups might not be high enough to reach statistical significance, we cannot rule out the possibility of inadequate participants and the follow-up time is not enough. Fourth, although statin usage is associated with reduction of all-cause mortality in current study, the true cause of death of many patients in both groups is not well defined; therefore, the explanation for reduction of all-cause death is still unclear. Fifth, overall interpretation may have been influenced by 3 factors: the absence of smoking status verification, the definition of ex-smokers, and the potential impact of passive smoking. All these variables could accentuate the detrimental effects of smoking by potentially elevating the risk of events among individuals classified as nonsmokers. Sixth, the verification of smoking status (e.g., through carbon monoxide measurement) was not conducted. This is noteworthy because there is often an underestimation of the number of smokers when comparing questionnaire responses to biochemically confirmed smoking status. Consequently, some individuals categorized as never-smokers or ex-smokers might have actually been active smokers. Moreover, there is a lack of information regarding the daily cigarette consumption. This data is pertinent as the number of years a person has smoked (‘smoking-years’) is a key indicator of heart disease risk associated with cigarette use. Finally, since the analysis of the current study is based on guideline-recommend statin therapy, whether statin usage could yield clinical benefits in current smokers undergoing PCI with a serum LDL level <100 mg/dL remains obscure.

## 5. Conclusion

Compared with persistent tobacco users undergoing PCI with average LDL-C level, the MACE rate seems not to be reduced in those with mildly elevated LDL-C level receiving statin therapy; nevertheless, statin therapy was still associated with a reduction of all-cause mortality.

## Acknowledgments

The authors wish to thank Chia-Chen Huang (PhD), Department of Public Health, Chung-Shan Medical University, Taiwan, for her assistance in statistical analysis.

## Author contributions

**Conceptualization:** Mao-Jen Lin, Han-Ping Wu.

**Data curation:** Hau-De Lin, Chuan-Zhong Cai, Ming-Jen Chuang, Feng-Ching Yang, Kuo Feng Chiang.

**Formal analysis:** Chuan-Zhong Cai.

**Funding acquisition:** Hau-De Lin.

**Investigation:** Mao-Jen Lin, Han-Ping Wu.

**Methodology:** Mao-Jen Lin.

**Project administration:** Han-Ping Wu.

**Resources:** Hau-De Lin, Chuan-Zhong Cai, Ming-Jen Chuang, Feng-Ching Yang, Kuo Feng Chiang.

**Software:** Ming-Jen Chuang.

**Supervision:** Han-Ping Wu.

**Validation:** Han-Ping Wu.

**Visualization:** Han-Ping Wu.

**Writing – original draft:** Mao-Jen Lin.

**Writing – review & editing:** Mao-Jen Lin, Han-Ping Wu.
